# Renal Manifestations in Scleroderma: Evidence for Subclinical Renal Disease as a Marker of Vasculopathy

**DOI:** 10.1155/2010/538589

**Published:** 2010-08-17

**Authors:** Victoria K. Shanmugam, Virginia D. Steen

**Affiliations:** Division of Rheumatology, Immunology and Allergy, Georgetown University Hospital, 3800 Reservoir Road, N.W, Washington, DC 20007, USA

## Abstract

Scleroderma is a disease characterized by immune activation, vasculopathy, fibroblast stimulation, and connective tissue fibrosis. End-organ damage occurs due to progressive tissue fibrosis and vasculopathy. Markers of incipient vasculopathy have not been well studied in scleroderma. However, reduced renal functional reserve and proteinuria are common indicators of progressive vasculopathy in diabetic and hypertensive vasculopathy. Recent studies suggest a strong association between renal involvement and outcomes in scleroderma, with a threefold increased risk of mortality from pulmonary hypertension if renal insufficiency is present. We review the types of renal involvement seen in scleroderma and the data to support the use of renal parameters including proteinuria, glomerular filtration rate, and renal vascular dynamics measured with Doppler ultrasound to identify subclinical renal insufficiency. Further studies are warranted to investigate the use of renal parameters as prognostic indicators in scleroderma.

## 1. Introduction

Systemic sclerosis (SSc) is a chronic multisystem disorder with an annual incidence of 1 to 2 per 100,000 individuals in the United States. It has a peak age of onset of between 30 and 50 years and a strong female predominance [1]. The exact pathogenesis of SSc remains elusive but, autoantibody production, lymphocyte and fibroblast activation, vascular proliferation, obliterative microvascular disease, and connective tissue fibrosis all likely play a role [2]. In advanced disease, end-organ damage occurs as a result of progressive fibrosis and vasculopathy. Often by the time organ injury is identified, there is little that can be done to reverse vasculopathy, and therefore there is a strong impetus in the scleroderma community to identify potential markers of incipient vasculopathy before damage becomes clinically apparent and irreversible.

Numerous other diseases associated with vascular damage, such as diabetes and hypertension, use markers of renal impairment as preclinical indicators of vasculopathy. In this paper we review the types of renal involvement reported in scleroderma and discuss preclinical markers of renal pathology that might be helpful in identifying scleroderma patients at risk for progressive vasculopathy.

## 2. Clinical Subsets of Scleroderma

Two subsets of scleroderma, with different autoantibody profiles and internal organ involvement are recognized [3]: limited cutaneous scleroderma (lcSSc) in which cutaneous involvement is limited to the hands, face, feet, and forearms and diffuse cutaneous scleroderma (dcSSc) in which there is extensive skin involvement extending above the elbows and knees and involving the trunk. A further group, scleroderma sine scleroderma (sSSc) in which patients have manifestations of visceral disease without skin involvement, has an identical prognosis to lcSSc and is included in the lcSSc group [3–5]. Patients with scleroderma sine scleroderma are often unaware of the disease until end-organ damage becomes apparent. These subsets are well described elsewhere. 

Patients with dcSSc develop rapidly progressive skin involvement with early organ involvement including interstitial lung disease, scleroderma renal crisis, and gastrointestinal involvement. One of the predictors of scleroderma renal crisis is the presence of the RNA polymerase III antibody (Pol3), and patients with this antibody are at high risk for early scleroderma renal crisis (SRC). In contrast, the anticentromere antibody is negatively associated with scleroderma renal crisis. Patients with limited scleroderma tend to have more indolent progression of skin disease but with time develop complications from vascular injury such as gastric antral vascular ectasia [6] and pulmonary hypertension.

## 3. Renal Disease in Scleroderma

Several forms of renal involvement are recognized in scleroderma. The most dramatic of these is scleroderma renal crisis which is seen in approximately 10% of the scleroderma population [7]. Autopsy studies, however, reveal occult renal pathology in 60% to 80% of patients with systemic sclerosis [8]. Others have found that up to 50% of asymptomatic patients have clinical markers suggesting renal disease such as proteinuria, elevation of creatinine, or hypertension [9]. Renal impairment from chronic renal vasculopathy, nephrotoxic medications (including cyclosporine and D-penicillamine), and glomerulonephritis have all been reported (Table 1) [10, 11]. 

### 3.1. Scleroderma Renal Crisis

**Figure 1:**
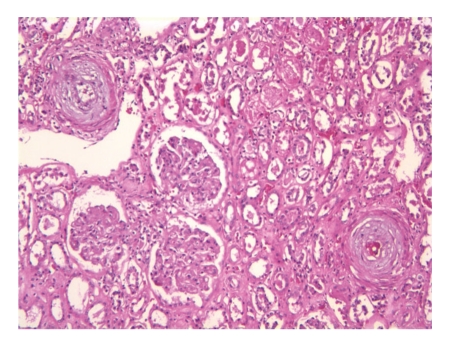
Hemotaxylin and Eosin stain of renal biopsy from a patient with scleroderma renal crisis, showing onion skinning concentric narrowing of arterioles with ischemia of glomeruli with flattening and degeneration of the tubular cells.

Scleroderma renal crisis (SRC) is one of the most well-recognized complications of scleroderma. It is manifested by acute onset of moderate-to-severe hypertension with hyperreninemia, thrombotic microangiopathy, and progressive renal failure [7]. Biopsy reveals severe mucinoid hyperplasia and vascular fibrinoid necrosis of the interlobular and arcuate arteries and arterioles (Figure 1) with relative sparing of the glomeruli and absence of inflammatory or immune deposits. With advanced renal crisis secondary ischemic changes in the glomeruli may occur. 

The pathologic findings seen in SRC suggest that it is an acute vascular manifestation of the disease. However, the renal vascular lesions may be present in SSc patients with normal renal function. Presence of these changes correlates with abnormal plasma rennin levels at baseline and in response to cold exposure but do not always correlate with development of SRC [12]. Risk factors for SRC include early (less than 5 years of disease) diffuse scleroderma, rapidly progressive skin thickening, presence of Pol3 antibody, and prior exposure to corticosteroids (>15 mg prednisone equivalent in the prior six months) [13]. Renal crisis is seen in approximately 10–20 percent of patients with dcSSc and only 1 percent of patients with lcSSc. However, of patients developing SRC, 78% had dcSSc [14]. Steroids are a precipitant in 60% of patients with SRC [14, 15]. Angiotensin converting enzyme inhibitors (ACE inhibitors) have been pivotal in the treatment of SRC, and since their introduction, mortality and renal morbidity has declined dramatically [16]. 

### 3.2. Normotensive Scleroderma Renal Crisis

A number of cases of acute, rapidly progressive renal failure with concomitant thrombotic microangiopathy but no malignant hypertension have been described, and this complication is termed “normotensive scleroderma renal crisis” [17, 18]. This manifestation is rare, accounting for only 11% of scleroderma renal crisis cases, and is associated with increased frequency of pulmonary hemorrhage and increased mortality. Anecdotal reports suggest that this presentation of renal crisis may be more common in patients receiving ACE inhibitors, but the mechanism by which this occurs is not well understood.

### 3.3. Coexistent MPO-ANCA-Associated Glomerulonephritis and Scleroderma

Several authors have reported myeloperoxidase antineutrophil cytoplasmic antibody- (MPO-ANCA-) associated glomerulonephritis resulting in renal failure in scleroderma [10, 19, 20]. In contrast to classical scleroderma renal crisis, these patients tend to have established lcSSc, and the process has a subacute presentation with progressive renal failure, mild hypertension, and proteinuria. This diagnosis should be considered in any scleroderma patient with positive MPO antibodies and renal failure. It has been postulated that scleroderma vasculopathy exacerbates the interaction of ANCA with endothelium near the vascular pole and neutrophil activation in the glomerulus. This manifestation does not respond to ACE inhibitors but is steroid responsive. However, biopsy is often required to exclude scleroderma renal crisis.

### 3.4. Penicillamine-Induced Renal Injury in Scleroderma

D-Penicillamine is rarely used now but historically has been used in treatment of Wilson's disease, cystinuria, rheumatoid arthritis, primary biliary cirrhosis, lead poisoning, and systemic sclerosis. Up to 20% of patients treated with D-penicillamine develop membranous glomerulopathy with proteinuria which resolves with cessation of the medication. Drug-induced lupus syndrome, diffuse proliferative cresentic glomerulonephritis, and cases of pulmonary renal syndrome mimicking Goodpasture's Syndrome have also been reported [21]. Typically these manifestations improve with withdrawal of the D-penicillamine, but in severe cases steroids, plasmapheresis, and immunosuppression have been required. Based on cases reported in the literature, this complication has a roughly 40% mortality.

### 3.5. Antiphospholipid-Associated Nephropathy in Scleroderma

Unlike in other autoimmune diseases, the frequency of antiphospholipid antibodies in scleroderma is reported to be no greater than that seen in the general population ranging from 3.3% to 12% [22–24]. Based on a single aPL antibody measurement of anticardiolipin (ACL) or Beta-2 glycoprotein I (B2GPI) antibody of IgM or IGG class, Wielosz et al. found that 56% of SSc patients had positive aPL antibodies [25]. They correlated antibody positivity with renal function parameters and found that although there was no difference in serum creatinine between the aPL positive group and the aPL negative group, IgG ACL was associated with elevation of serum cystatin C (a marker of reduced glomerular filtration rate) and negatively associated with creatinine clearance. Furthermore, proteinuria (>0.5 g/24 hours) was found in 21% of the APL positive patients but only in 9% of the APL negative patients. aPL positivity was also associated with development of SRC, which occurred in 21% of the patients with positive aPL and none of the patients with negative aPL. Larger studies are needed to clarify these findings, but this suggests that aPL antibodies may play a role in renal injury in scleroderma.

### 3.6. Isolated Reduced Glomerular Filtration Rate in Scleroderma

Impairment of renal function can be present in SSc despite normal serum creatinine [26]. The principal determinants of creatinine are muscle mass and glomerular filtration rate (GFR), and it is well recognized that serum creatinine may not be elevated until the GFR is less than 50% of normal. Kingdon et al. evaluated patients followed in the Royal Free Hospital Scleroderma Clinic and found reduced GFR as measured by chromium-51-ethylenediaminetetraacetic acid (51Cr-EDTA) clearance in 95% of patients with serum creatinine within the normal range and all the patients with elevated creatinine. These patients also had abnormal calculated GFR. In patients with serum creatinine below the normal range (<60 *μ*mol/L) the measured GFR was normal. The highest correlation between measured and calculated GFR was seen when the Modification of Diet in Renal Disease (MDRD) formula was used. This group advocates using MDRD to calculate GFR at baseline and followup visits. 

More recently Scheja et al. have tried to investigate the occurrence of reduced GFR in a large cohort of 461 Swedish SSc patients followed over 10 years [27]. They measured GFR using either chromium-51-ethylenediaminetetraacetic acid (51Cr-EDTA) or by iohexol clearance. Patients with a history of SRC or who developed SRC during follow-up were excluded from analysis. Median follow up was 7.7 years (range 0.5–54 years). Decreased GFR was found in 11% of lcSSc and 8.6% of dcSSc, which accounts for approximately 10% of the total initial cohort. This is lower than reported in the Royal Free study but may simply reflect differing inclusion criteria. The Swedish study included 461 consecutive SSc patients; in contrast the Royal Free study included only 26 patients who were selected based on availability of measured and calculated GFR results on two occasions, an inclusion criteria which is likely to bias the results towards overrepresentation of patients with abnormal GFR. Of the patients with GFR <70% predicted in the Swedish cohort, 60% had hypertension, 52% had cardiac involvement, and 19% had other nephropathies identified on biopsy. Follow up data beyond 4 years was only available in 15 patients, but the majority (73%) had no progression of renal disease in that time. 

Steen et al. examined records of 675 patients with dcSSc, and after excluding the 19.5% who developed SRC, they found 32% with abnormal renal function or proteinuria [28]. Most patients had proteinuria from penicillamine or other medical comorbidities. Only 2% had no explanation for elevated creatinine level. Over a mean follow-up of 10 years, none of these patients developed renal failure requiring dialysis, suggesting that although mild renal insufficiency is common in SSc, it often follows a more benign clinical course. 

A smaller study from Cairo has evaluated 31 SSc patients and compared them to 31 healthy controls [29]. GFR was measured using technetium 99m DTPA (Tc99mDTPA) and calculated using MDRD and Cockcroft-Gault formulae. All patients had normal serum creatinine and normal renal ultrasound. Measured GFR was normal (>89 ml/min) in 45.1% but reduced in 54.9% as follows: Stage II CKD 60–89 ml/min 32.3%, Stage III (30–59 ml/min) in 22.6%. Renal impairment correlated to pulmonary vascular involvement but did not correlate with age, disease duration, lung fibrosis, gastrointestinal involvement, cardiac involvement, skin score, muscle involvement, antibody profile, or treatment exposures. 

The association between renal dysfunction and pulmonary hypertension in SSc is increasingly becoming recognized. Campo et al. recently evaluated 76 consecutive SSc patients with pulmonary arterial hypertension (PAH) and found that 45.6% had renal dysfunction (eGFR <60 mL/min/1.73 m^2^) at the time of diagnosis despite only 6.5% having had a prior episode of renal crisis [30]. Furthermore, eGFR was a strong predictor of survival in this cohort, with eGFR <60 mL/min/1.73 m^2^ associated with a 3-fold risk of mortality. This strong association may be a reflection of pulmonary hypertension and right heart failure contributing to renal dysfunction through fluid retention and neuroendocrine activation. However, further studies are warranted to evaluate the role of renal dysfunction in SSc-associated pulmonary hypertension.

### 3.7. Reduced Renal Functional Reserve in Scleroderma

Renal functional reserve is a measure of the kidney's ability to increase GFR after stimulation with oral protein or IV amino acid load. Livi et al. studied renal functional reserve in 21 SSc patients (16 with lcSSc and 5 with dcSSc) with normal renal function and compared them to 10 control patients [31]. Effective renal plasma flow (ERPF) using para-aminohippurate clearance and calculated total renal vascular resistance (TRVR) were measured before and after an intravenous amino acid load. Creatinine clearance was similar at baseline in the two groups. However, the ERPF response was significantly lower, and TRVR was higher in patients than controls. In normal subjects, GFR should rise by 10% in response to stimulation, but only 28.6% of the SSc patients showed this response. Blunted renal functional reserve was seen in 80% of patients with dcSSc and 68.75% of patients with lcSSc. Additional studies are needed to establish if lack of renal functional reserve in SSc is a predictor of developing clinically evident renal involvement or vasculopathy in other organs.

### 3.8. Proteinuria in Scleroderma

Albuminuria is a useful marker of vasculopathy and is known to be an independent predictor of cardiovascular morbidity and mortality in patients with and without other vasculopathic diseases such as diabetes and hypertension [32–36]. Seiberlich et al. analyzed urine albumin, urine total protein, and urine electrophoresis to assess protein excretion in 80 SSc patients and 18 healthy age- and gender-matched controls [37]. All subjects had a normal GFR. Increased total protein excretion was seen in 17.5% of SSc patients, and albuminuria was identified in 25% (22.5% microalbuminuria and 2.5% macroalbuminuria). Albuminuria correlated with disease duration >4 years and elevation of systolic blood pressure, suggesting it may be reflective of chronic vascular injury. Dawnay et al. evaluated urine albumin in a cohort of scleroderma patients and found prevalence of microalbuminuria of 17.9% but this could not be correlated with clinical outcome due to the small sample size [38]. Larger studies are needed to evaluate the role of albuminuria as a predictor of morbidity, mortality, and outcome in scleroderma. 

Urine electrophoresis has not been widely studied in the scleroderma population. In general, urine electrophoresis results are categorized into three patterns depending on the molecular weight of the protein detected. Low molecular weight proteinuria (LMWP) is usually due to diminished tubular resorptive capacity for example from interstitial nephritis and nephrotoxicity. In the Seiberlich study, LMWP was seen at a similar frequency in the SSc group as controls [37]. Intermediate weight proteinuria (IMWP) is a sensitive predictor of increased glomerular permeability and has been described in other forms of vasculopathy including diabetes and hypertension [39]. Urine electrophoresis in the Seiberlich study revealed IMWP in 31.3% of SSc patients, but none of the control subjects. IMWP was seen in 50% of patients with dcSSc compared to only 20% of lcSSc and correlated to the presence of gastrointestinal involvement. Francois et al. have also reported an association of glomerular proteinuria with scleroderma but did not correlate this to clinical parameters [40]. High molecular weight proteinuria (HMWP) is generally a reflection of glomerulonephritis and was not seen in any of the SSc patients or controls in the Seiberlich study. 

Further studies are warranted to investigate the role of proteinuria and albuminuria detection in the scleroderma population. In diabetic vasculopathy it is recommended that initiation of angiotensin converting enzyme (ACE) inhibitors when microalbuminuria is detected is effective at delaying progression to advanced disease and improves cardiovascular outcomes [41]. While ACE inhibitors are known to exhibit antifibrotic effects [42] in addition to lowering systemic blood pressure, the use of prophylactic ACE inhibitors is generally not recommended in SSc. Use of prophylactic ACE inhibitors in SSc does not protect against SRC [15, 43], and their use prior to development of SRC may be associated with worse outcomes [14, 44] although this data was not adjusted for confounders, and the reported association did not reach statistical significance.

### 3.9. Renal Vascular Resistance Indices in Scleroderma

With the advent of new imaging techniques, several groups have investigated the use of non-invasive testing such as renal Doppler ultrasound to evaluate intrarenal vasculature in scleroderma patients. 

In patients with lupus nephritis the renal vascular resistance index (RI) is a predictor of poor outcome, correlating with creatinine level and chronicity score on biopsy [45]. RI measures intrarenal elasticity and compliance and tends to be more sensitive to vascular and interstitial nephropathies because glomeruli are only responsible for 10% of the intraparenchymal flow resistances. Release of vasoconstrictive substances results in renal remodeling and elevation of RI as renal vascular disease progresses. In hypertension, both RI and albuminuria improve with angiotensin receptor blocking (ARB) agents [46], suggesting that RI may be measuring a potentially reversible component of vasculopathic damage. 

Investigating SSc patients without clinical evidence of renal damage, Rivolta et al. measured RI on the main, interlobar, and cortical vessels in 25 SSc subjects and 25 normal volunteers. SSc patients had significantly elevated RI at every sampling site [47]. RI values correlated with disease duration but not creatinine clearance. These findings have since been replicated in a study evaluating 9 SSc patients with renal impairment, 13 SSc patients with normal renal function, and 20 age-matched controls. There was no significant difference in peak systolic flow velocities in any of the investigated arteries, between patients and control groups. However, the mean end diastolic flow velocity (EDFV) in the interlobular artery (ILA) was lower in the SSc patients with renal disease compared to the SSc patients without renal involvement. SSc patients with renal disease had higher systolic to diastolic flow velocities (S/D ratio). The mean RI and pulsatile index (PI) were higher in SSc patients with renal disease than those without. RI, PI, and S/D ratios correlated with disease duration but were over and above those that might have been expected with age. Further studies are warranted to evaluate the use of Doppler ultrasound in predicting renal complications in scleroderma.

### 3.10. Renal Vascular Responsiveness to Iloprost in Scleroderma

Further understanding of the mechanisms of vascular involvement in SSc is obtained by evaluating the response of renal and other vascular beds to vasodilating agents. 

In normal tissue, prostacyclin is released locally from platelets and vascular endothelium and inhibits production of endothelin, a potent vasoconstrictor. SSc patients have elevated levels of endothelin I and are known to be prone to toxicity from cyclosporine, an agent that causes vasoconstriction and elevation of endothelin I. Furthermore, there is some evidence that endothelin I may play a role in SRC [48]. 

Iloprost is a stable prostacyclin analogue with vasodilatory and platelet aggregating effects. It is used in Raynaud's syndrome and is effective in healing cutaneous scleroderma lesions. It has long lasting effects on peripheral blood flow and improves pulmonary artery resistance in PHT. In the kidney it increases renal plasma flow by dilating afferent and efferent arterioles, without changing GFR, and some centers recommend including intravenous iloprost during the hypertensive phase of SRC [44]. 

Scorza et al. investigated the effect of iloprost on RI in the renal vessels and compared it to the calcium channel antagonist nifedipine [49]. They found that acute iloprost infusion resulted in significant drop of RI in the interlobar and cortical vessels without causing systemic hypotension. Use of iloprost for 8 hours per day for 5 days had long-lasting effect on reducing the RI values in the interlobular and cortical arteries at 2 weeks. In contrast, 6 months of nifedipine treatment did not alter the RI of the renal vessels. 

Long-term renal outcomes in response to iloprost therapy remain to be elucidated. Airò et al. evaluated the use of cyclic iloprost in a cohort of SSc patients with severe digital ulcers and Raynaud's and compared pulmonary and renal outcomes over 4 years with age, sex- and disease-matched controls [50]. There was no detectable difference in pulmonary, renal, or long-term outcomes in this study but selection bias and other confounding may have affected these results.

### 3.11. N-Acetylcysteine Infusion in Early Scleroderma

Renal hemodynamics in SSc patients have also been studied in response to N-Acetylcysteine (NAC) [51]. NAC is a sulfhydryl precursor of glutathione with potent antioxidant and cellular detoxifying actions. Via vasodilation and actions on oxygen free radical scavenging, NAC has been show to improve myocardial function after myocardial infarction [52]. In the kidney, NAC has been shown to enhance renal glutathione, ameliorate renal function, decrease arterial pressure and renal injury in salt sensitive hypertension, and improve endothelial dysfunction in dialysis patients by preventing flow-mediated dilatation [53, 54]. In radiocontrast exposure, it has an antioxidant function and causes vasodilatation and enhanced renal medullary flow [55]. 

In an open-label study [51], 40 SSc patients with either early or late capillaroscopic changes received NAC intravenously for 5 hours at 0.015 g/Kg/h, and renal hemodynamics were assessed using RI. In patients with early capillaroscopic changes, NAC reduced RI. In contrast, in the group with late capillaroscopic pattern, RI was increased. Based on these findings, it appears that NAC results in renal arterial vasodilation only if given early in the course of disease, prior to irreversible arteriopathy. Patients in this study with late capillaroscopic changes all had low diffusion capacity for carbon monoxide (DLCO), suggesting that they had a component of pulmonary vascular involvement. It seems that as vasculopathy becomes more extensive, NAC may have a detrimental effect, and the implications of this for the use of NAC clinically are unclear.

### 3.12. Vascular Endothelial Markers in Scleroderma Renal Disease

Cytokine activation of endothelial cells in states of inflammation induces expression of adhesion molecules that allow recruitment of leukocytes to the sites of inflammation [56, 57]. Activated, endothelial cells shed soluble forms of these adhesion molecules including intracellular adhesion molecule-1 (ICAM1), vascular cell adhesion molecule-1 (VCAM1), and E-selectin (E-selectin), and these molecules can be detected in the serum. It is thought that E-selectin has the greatest specificity for cytokine-activated cells. Prior studies have shown elevation of these molecules in diseases with endothelial injury including SLE, vasculitis, and sepsis [58, 59]. 

Stratton et al. evaluated levels of E-selectin, ICAM-1, and VCAM-1 in serum of SSc patients with and without SRC and SSc-associated pulmonary hypertension (PHT) and compared them to control patients with primary Raynaud's [60]. They found that E-selectin was elevated approximately 25% above normal in patients with lcSSc without SRC or PHT. In contrast, E-selectin levels were normal in patients with lcSSc and PHT, suggesting that the vascular lesions of PHT were not associated with activated endothelial cell phenotype. Although SRC is rare in lcSSc, E-selectin levels were two times normal in the lcSSc-SRC group, suggesting that there are different pathogenic mechanisms of vascular injury in SRC and PHT. Notably, all patients with dcSSc and PHT (all of whom had concomitant interstitial disease) did show elevation of E-selectin, suggesting that inflammatory lung disease leads to activation of endothelial cells in the pulmonary microcirculation with increased expression and shedding of E-selectin. 

Serum VCAM-1 levels were elevated in both lcSSc and dcSSc patients with SRC. They were also increased in patients with lcSSc without SRC or PHT, suggesting they may be a reflection of the chronic vascular injury in this subgroup of patients. Soluble ICAM-1 levels were raised in all groups with the highest levels being seen in dcSSc with PHT. Analysis of serial measurements did not show consistent trends or progressive increase in endothelial markers over time. Likewise, in patients with SRC, endothelial markers were not consistently elevated at the time of SRC.

## 4. Conclusions

Mild chronic renal insufficiency in scleroderma is probably under recognized and may be a manifestation of vasculopathy. Recent studies suggest a strong association between renal involvement and outcomes in scleroderma, with a threefold increased risk of mortality from pulmonary hypertension if renal insufficiency is present. Current data suggests that manifestations of renal insufficiency including proteinuria and altered renal vascular dynamics as measured with Doppler ultrasound may help identify early signs of renal involvement, and this may be a surrogate marker of vasculopathy. Further studies are warranted to investigate renal markers as prognostic indicators and targets for disease modifying therapy in scleroderma.

## Figures and Tables

**Table 1:** Reported renal manifestations of scleroderma.

Reported renal manifestations of scleroderma
Scleroderma renal crisis
Normotensive scleroderma renal crisis
Myeloperoxidase-Antineutrophil Cytoplasmic Antibody (MPO-ANCA) associated glomerulonephritis and vasculitis
Penicillamine-associated renal disease
Antiphospholipid-associated nephropathy
Isolated reduced glomerular filtration rate
Reduced renal functional reserve
Microalbuminuria and proteinuria
Scleroderma-associated vasculopathy manifested by abnormal renal vascular resistance indices and endothelial markers
